# The workday of hospital surgeons: what they do, what makes them satisfied, and the role of core tasks and administrative tasks; a diary study

**DOI:** 10.1186/s12893-019-0570-0

**Published:** 2019-08-14

**Authors:** Eliane Holzer, Franziska Tschan, Maria U. Kottwitz, Guido Beldi, Adrian P. Businger, Norbert K. Semmer

**Affiliations:** 1 Institute for Work and Organizational Psychology, University of Neuchâtel, Rue Emile-Argand 11, 2000 Neuchâtel, Switzerland; 2 Department of Psychology, University of Bern, Fabrikstr. 8, 3012 Bern, Switzerland; 3 Department of Visceral Surgery and Medicine, University Hospital of Bern, Bern, Switzerland; 4 Federal Department of Defense, Swiss Armed Forces, Switzerland and Private University in the Principality of Liechtenstein, Triesen, Liechtenstein; 5 Department of Psychology, University of Marburg, Marburg, Germany

**Keywords:** Hospital surgeons, Daily activities, Task attractiveness, Core tasks, Administration, Job satisfaction

## Abstract

**Background:**

Many surgeons report passion for their work, but not all tasks are likely to be satisfying. Little is known about how hospital surgeons spend their days, how they like specific tasks, and the role of core tasks (i.e. surgery-related tasks) versus tasks that may keep them from core tasks (e.g., administrative work). This study aimed at a more detailed picture of hospital surgeons’ daily work - how much time they spend with different tasks, how they like them, and associations with satisfaction.

**Methods:**

Hospital surgeons (*N* = 105) responded to a general survey, and 81 of these provided up to five daily questionnaires concerning daily activities and their attractiveness, as well as their job satisfaction. The data were analyzed using t-tests, analysis of variance, as well as analysis of covariance and repeated measures analysis of variance for comparing means across tasks.

**Results:**

Among 14 tasks, surgery-related tasks took 21.2%, patient-related tasks 21.7% of the surgeons’ time; 10.4% entailed meetings and communicating about patients, and 18.6% documentation and administration. The remaining time was spent with teaching, research, leadership and management, and not task-related activities (e.g. walking between rooms). Surgery was rated as most (4.25; *SD* = .66), administration as least attractive (2.63; *SD* = .78). A higher percentage of administration predicted lower perceived legitimacy; perceived legitimacy of administrative work predicted job satisfaction (*r* = .47). Residents were least satisfied; there were few gender differences.

**Conclusions:**

Surgeons seem to thrive on their core tasks, most notably surgery. By contrast, administrative duties are likely perceived as keeping them from their core medical tasks. Increasing the percentage of medical tasks proper, notably surgery, and reducing administrative duties may contribute to hospital surgeons’ job satisfaction.

**Electronic supplementary material:**

The online version of this article (10.1186/s12893-019-0570-0) contains supplementary material, which is available to authorized users.

## Background

Many surgeons see their profession as fascinating, citing feelings of passion as a reason for being a surgeon [1], or referring to surgery as a calling [2]. At the same time, about a fifth of hospital surgeons think about giving up their profession several times a month [3]. Furthermore, the prevalence of depression and burnout among surgeons is rather high, and so is suicide [4, 5].

Such problems are likely connected with high workload and long working hours [6–8] and the related issues of high fatigue/exhaustion [6] (the main component of burnout), and with an impaired balance between work and private life [2, 4, 6, 7].

Workload and stress levels are higher than in other medical specialties [3, 7]. The high stress levels can contribute to burnout [9], which has been shown to be associated with lower satisfaction with various aspects of the work situation in a variety of studies [4, 10–12]. Surgeons do indeed report lower job satisfaction than other physicians [13].

To some extent, however, such problems might be associated with the nature of the tasks themselves. Specifically, tasks that are not properly surgical and not directly related to patient care, most notably a high percentage of administrative work, are often cited as reasons for stress and dissatisfaction [2, 7, 14–16]. These findings are mainly based on studies that represent rather general evaluations of the work situation; much less is known about the daily life of surgeons. To tailor attempts at optimizing conditions to the specific circumstances at work, we need a more detailed picture of what the daily working life of surgeons looks like, what they actually do, and what they like most and least about their work. In this study, hospital surgeons responded to a number of daily questionnaires in addition to a general survey. Such a diary approach is less prone to bias by errors of memory than general surveys [17].

## Methods

### Recruitment and participants

The research team sent an information letter to public hospitals in the German-speaking part of Switzerland, explaining the purpose of the study and the process of data collection. Of 71 hospitals contacted, 26 agreed to inform their surgeons, 11 refused to participate, and 34 did not respond. Surgeons from 22 different hospitals participated in the study. Classified according to the Swiss Medical Association (http://www.siwf-register.ch), one of the participating hospitals (4.6%) was a University hospital, 9 (40.9%) were primary referral centers (type A, offering 4 years of surgical training), 4 (18.8%) were secondary referral hospitals (type B3, offering 3 years of training) and 8 (36.4%) were small regional surgical departments (one type B1; seven B2, offering one or 2 years of training). Thus, type A hospitals were overrepresented (40.9% versus 19.2%), and B1 hospitals were underrepresented (4.6% versus 19.2%).

In 18 participating hospitals, the research team directly presented the study at the hospital; for four hospitals, the information was provided by electronic mail. After being informed about the study, 132 surgeons signed up for participation and received a detailed information package. Subsequently, 27 surgeons withdrew their agreement – two because of lack of time, one because of imminent retirement; 24 did not provide a reason.

The final sample consists of 105 surgeons; 40 (38.1%) were female. Mean age was 37.4 years (*SD* = 10.46, *RANGE* = 24–64). Positions were chief of staff (14; 13.33%), consultant (13; 12.38%), Fellow/chief resident (30; 28.57%), and resident (48; 45.71%). Mean tenure in the current hospital was 4.49 years (*SD* = 6.18; *RANGE* 0–30). With reference to the 132 surgeons who initially had agreed to participate, the response rate was 79.5% (105/132) for the general questionnaire, and 61.4% (81/132) for the daily questionnaires.

Overall, 81 surgeons provided 374 daily records, reporting data on 4.6 days on average (67 for 5 days; 7 for 4, 1 for 3; 2 for 2, and 4 for 1). Excluding daily records that did not specify the time spent on specific tasks resulted in 338 daily records included in the analyses.

### Procedure

Questionnaires were delivered via e-mail. The general survey contained demographic questions and general questions about the participants’ work, not all of which are reported here. After completing the survey, participants provided five dates during which they worked at the hospital and could respond to daily questionnaires; on each of these they received an email in the morning containing a personalized link to the questionnaire of the day. We recommended answering the daily questionnaires late afternoon, because typically no regular surgeries are scheduled at this time. Questionnaires were presented online, using Qualtrics (Provo, UT).

### Measures

#### General survey

The general survey asked about demographics (age, sex, position, etc.), and about general working conditions. Concerning the latter, job satisfaction and perceived opportunities for training are reported in this paper. General job satisfaction was assessed with an item ranging from 1 (exceptionally dissatisfied) to 7 (exceptionally satisfied); the verbal descriptions were combined with faces that look more or less satisfied [18]. Training opportunities were assessed with four questions of the scale “continuing education and training” from the Instrument for Stress-Oriented Task Analysis for Hospital Physicians [19], such as “in our department, inexperienced medical colleagues have sufficient opportunity to profit from skills and knowledge of the more experienced ones”. Answers ranged from 1 (not true) to 5 (completely true); reliability (Cronbach’s alpha) was α = 0.85.

### Daily questionnaire

For each of 14 tasks (plus a category “other”; see Table 1) the surgeons specified whether they had performed the task within the last 24 h (no, once, several times), how much time they had spent on each task (hours; minutes), and how attractive they perceived the task to be on a 5 point Likert Scale ranging from 1 (very negative) to 5 (very positive). These task categories were developed based on pilot interviews with 50 surgeons. To assess whether surgeons considered a task as a legitimate part of their duties, it was also asked if a task made sense and whether they considered it necessary and reasonable that *they* carried out this task, based on the Bern Illegitimate Tasks Scale (BITS; [20]); the answering format was a 5 point Likert scale ranging from 1 (absolutely not) to 5 (very much).

Regarding administrative duties, we asked two questions developed for this study concerning perceived adequacy (“do you think the demand for administrative work in the last 24 hours overall a) was adequate, b) kept you from important medical activities?”; answers ranged from 1 = not true to 5 = very true); the two questions were combined; Cronbach’s alpha for this score was .83.

Current job satisfaction was assessed each day with the question "Regarding my situation at work overall, at his moment I am … extremely dissatisfied (1) to extremely satisfied (7); verbal answers were combined with faces as in the general questionnaire [18].

The questions that were developed specifically for this project are listed in an (Additional file 1).

### Analyses

Daily questionnaire data were aggregated within individual surgeons. Means and standard deviations are reported for numerical data, counts and percentages for categorical data. Using SPSS 21.0 [21], we analyzed data by t-tests and analysis of variance; when appropriate, we controlled for covariates using Analysis of Covariance (ANCOVA). Repeated measures analysis of variance with Bonferroni correction was used for comparing means across tasks, Tukey’s HSD test for post hoc analyses. We considered *P* < .05 as significant for all analyses.

## Results

### How surgeons spend their days

#### Differences between hospitals

There were no significant differences between hospitals with regard to sex, position, job satisfaction and satisfaction with responsibility during surgery, nor with regard to attractiveness or legitimacy of tasks. Differences that did emerge are mentioned below.

#### Time spent performing specific tasks

As shown in Table 1, participants spent approximately 2 ½ hours with surgery-related tasks (doing or preparing surgery). Another 2 ½ hours were spent with individual patient work, 1¼ hours with meetings and team communication about patients; somewhat less than an hour (47 min) with continuing education, teaching and research; approximately 27 min with management and leadership, and approximately 2¼ hours with documentation and administration (see Table 1 for the exact values).

**Table 1:** Time spent performing specific tasks and their perceived attractiveness

Tasks	Time spent with task				How much they liked doing the task (1 (very negative) - 5 (very positive))				
	Overall *N* = 78	Chief of staff/con- sultant *N* = 23	Fellow/chief resident *N* = 22	Resident *N* = 33	Overall	Chief of staff/consultant	Fellow/chief resident	Resident	
	M (SD)	M (SD)	M (SD)	M (SD)	M (SD) N	M (SD) N	M (SD) N	M (SD) N	
Surgery-related									
Performing surgery / assisting surgery	2h20m (1h4m)	2h38m (1 h23 m)	2h41m (1h50m)	1 h53 m (1h48m)	4.25 (.66) *N* = 68	4.28 (.44) *N* = 22	4.34 (.67) *N* = 22	4.13 (.81) *N* = 24	
Preparing surgical strategy	13 m (14 m)	14 m (13 m)	16 m (16 m)	10 m (13 m)	4.06 (.60) *N* = 58	4.18 (.57) *N* = 19	4.11 (.54) *N* = 20	3.89 (.69) *N* = 19	
Individual patient work									
Medical consultations*	1h10m (1 h)	1h31m (57 m)	1h7m (50 m)	58 m (1h6m)	3.76 (.59) *N* = 63	4.02 (.34) *N* = 20	3.92 (.51) *N* = 19	3.41 (.65) *N* = 24	*residents rated it as less attractive than the others
Ward Rounds*	58 m (41 m)	43 m (21 m)	45 m (25 m)	1 h16 m (52 m)	3.83(.60) *N* = 74	4.04 (.52) *N* = 23	3.96 (.50) *N* = 22	3.57 (.65) *N* = 29	*residents spent more time than chiefs/consultants and fellows/ chief residents and rated it as less attractive than others; women rated it as more attractive than men
Patient-related conversations*	25 m (20 m)	25 m (21 m)	17 m (13 m)	29 m (23 m)	3.58 (.65) *N* = 66	3.89 (.49) *N* = 19	3.67 (.50) *N* = 19	3.31 (.74) *N* = 28	*residents rated it as less attractive than the others
Meetings / communication about patients									
Daily meetings	45 m (23 m)	39 m (17 m)	45 m (25 m)	49 m (24 m)	3.53 (.67) *N* = 76	3.62 (.60) *N* = 23	3.5 (.64) *N* = 22	3.49 (.74) *N* = 31	
Specific meetings* (e.g., tumorboard)	14 m (14 m)	17 m (15 m)	13 m (11 m)	13 m (17 m)	3.63 (.78) *N* = 48	3.88 (.68) *N* = 18	3.42 (.77) *N* = 14	3.53 (.87) *N* = 16	*women rated it as more attractive than men
Other discussions about patients*	16 m (18 m)	16 m (16 m)	17 m (18 m)	15 m (20 m)	3.56 (.72) *N* = 50	3.96 (.63) *N* = 15	3.54 (.56) *N* = 17	3.24 (.79) *N* = 18	*residents rated it as less attractive than chiefs/consultants; women rated it as more attractive than men
Teaching / Research									
Continuing education	16 m (25 m)	18 m (31 m)	12 m (11 m)	16 m (27 m)	4.05 (.76) *N* = 44	4.4 (.53) *N* = 13	4.02 (.66) *N* = 15	3.79 (.93) *N* = 16	*residents rated it as less attractive than chiefs/consultants. Interaction position x gender: male residents rated it as less attractive than male fellows/chief residents, female residents rated it as more attractive than female fellows/chief residents.
Research	17 m (40 m)	12 m (33 m)	22 m (41 m)	17 m (43 m)	3.89 (.65) *N* = 24	4.08 (.66) *N* = 6	3.89 (.80) *N* = 10	3.75 (.46) *N* = 8	
Teaching*	14 m (25 m)	20 m (26 m)	23 m (32 m)	5 m (12 m)	4.22 (.50) *N* = 42	4.32 (.47) *N* = 17	4.17(.46) *N* = 16	4.13 (.62) *N* = 9	*residents spent less time than fellows/chief residents
Leadership and Management*	27 m (55 m)	1 h14 m (1h20m)	18 m (26 m)	0 m (0 m)	3.81 (.63) *N* = 34	3.81 (.56) *N* = 19	3.81 (.76) *N* = 14	4 (.00) *N* = 1	*residents spent less time than chiefs/consultants, who spent more time than fellows/chief residents
Documentation and Administration									
Patient documentation *	1 h25 m (1 h05 m)	51 m (36 m)	1 h (39 m)	2h06m (1 h13 m)	2.96 (.67) *N* = 72	3.19 (.45) *N* = 22	3.14 (.65) *N* = 21	2.66 (.72) *N* = 29	*residents spend more time than chief/consultants and fellows/chief residents; women spent more time than men; residents rated it as less attractive than the others
General Administration*	46 m (36 m)	42 m (30 m)	40 m (37 m)	53 m (39 m)	2.63 (.78) *N* = 72	2.88 (.67) *N* = 21	2.79 (.85) *N* = 22	2.32 (.70) *N* = 29	*residents rated it as less attractive than the others; women rated it as more attractive than men
Other^a^									
Related to medical tasks (e.g. walking between rooms, waiting)	2h2m (2h2m)	1 h33 m (1h54m)	2h40m (2h11m)	1h58m (1h59m)					
Unrelated to medical tasks (e.g. breaks, private calls)	5 m (13 m)	9 m (17 m)	3 m (13 m)	3 m (11 m)					

An asterisk in the first column indicates significant differences between groups; these are described in the last column

^a^The two subcategories are based on coding the answers provided

Except for time spent for research, which was more in larger hospitals, there were no significant differences between hospitals with regard to time spent for specific tasks. With regard to hospital type, surgeons in university hospitals spent more time for preparing surgery and for doing research and less time for reports.

#### Position differences

Significant differences in time spent on tasks between the positions concerned (a) ward rounds *F* (2, 73) = 4.42, *p =* .015, *η*^*2*^ = .11, with residents spending more time doing ward rounds than chiefs/consultants (*p* = .006) and fellows/chief residents (*p* = .013); (b) teaching *F* (2, 73) = 3.78, *p* = .027, *η*^*2*^ = .09, which was hardly done by residents; (c) leadership, *F* (2, 73) = 12.80, *p* < .001, *η*^*2*^ = .26, which was not done at all by residents, and (d) patient documentation, *F* (2, 73) = 11.42, *p* < .001, *η*^*2*^ = .24, with residents doing more patient documentation than both chief/consultants and fellows/chief residents (both: *p* < .001).

Controlling for position, only patient documentation showed a gender effect, *F* (1, 73) = 4.13, *p* = .046, *η*^*2*^ = .054, with women spending more time on this task than their male colleagues.

#### Attractiveness of tasks

Columns 6–9 of Table 1 reveal that the surgeons perceived tasks directly related to surgery as very attractive. Teaching and continuous education also received high ratings. By contrast, administrative task received the lowest rating by all groups. More specifically, repeated measures ANOVA showed that the task that is liked most is performing surgery; it is rated as more attractive than eight other tasks (administration, patient documentation, patient related discussions [including calls from general practitioners], daily meetings, special meetings, consultation hours, ward rounds). Teaching is next, which is liked significantly more than seven other tasks. The two tasks that were liked the least were administration and patient documentation, which were perceived as significantly less attractive than all the other tasks. Whenever there were significant differences between positions, it was residents who rated the respective task as less attractive than the others. Whenever gender differences were found, it was women who rated the respective task as more attractive than men. Male residents evaluated further training as less attractive than male fellows/chief residents, but female residents evaluated further training as more attractive than female fellows/chief residents.

The attractiveness of almost all tasks was correlated with the attractiveness of several other tasks, with significant correlations ranging from *r* = 0.256 to *r* = 0.755. Doing research was the only task the attractiveness of which was not correlated with the attractiveness of any other task.

#### Administrative tasks and the issue of legitimacy

As described above, participants noted in the daily questionnaires if they considered that a task they carried out made sense, and whether it was necessary and reasonable that *they* carried out this task, indicating the perceived degree of legitimacy [22]. A repeated measures ANOVA showed that surgery-related tasks were rated as more legitimate than five other tasks, making them the tasks that were perceived as most legitimate of all (mean legitimacy = 4.48 for surgery, 4.53 for preparing for surgery). Administration was rated as significantly less legitimate than every other task, followed by writing patient documentation, which was rated as less legitimate than six other tasks (mean administration = 3.12, mean patient documentation = 3.68).

**Fig. 1:**
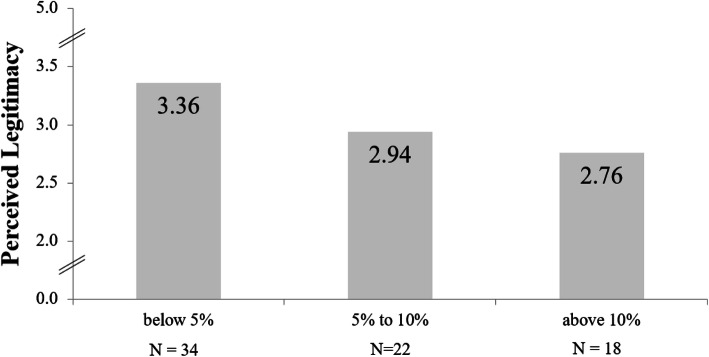
Amount of administrative work and its perceived legitimacy

We also asked two questions about the amount of administrative duties over the workday in terms of their perceived legitimacy (see Methods section). The combined value of these two items was correlated with the proportion of time spent with administrative duties at *r* = − 0.313, *p =* .007. Thus, as the proportion of administrative tasks increases, their perception as being inadequate and keeping one from important medical tasks increases as well, indicating low legitimacy. Figure 1 shows how the values for perceived legitimacy of administrative work decrease as its proportion increases.

#### Daily satisfaction

**Fig. 2:**
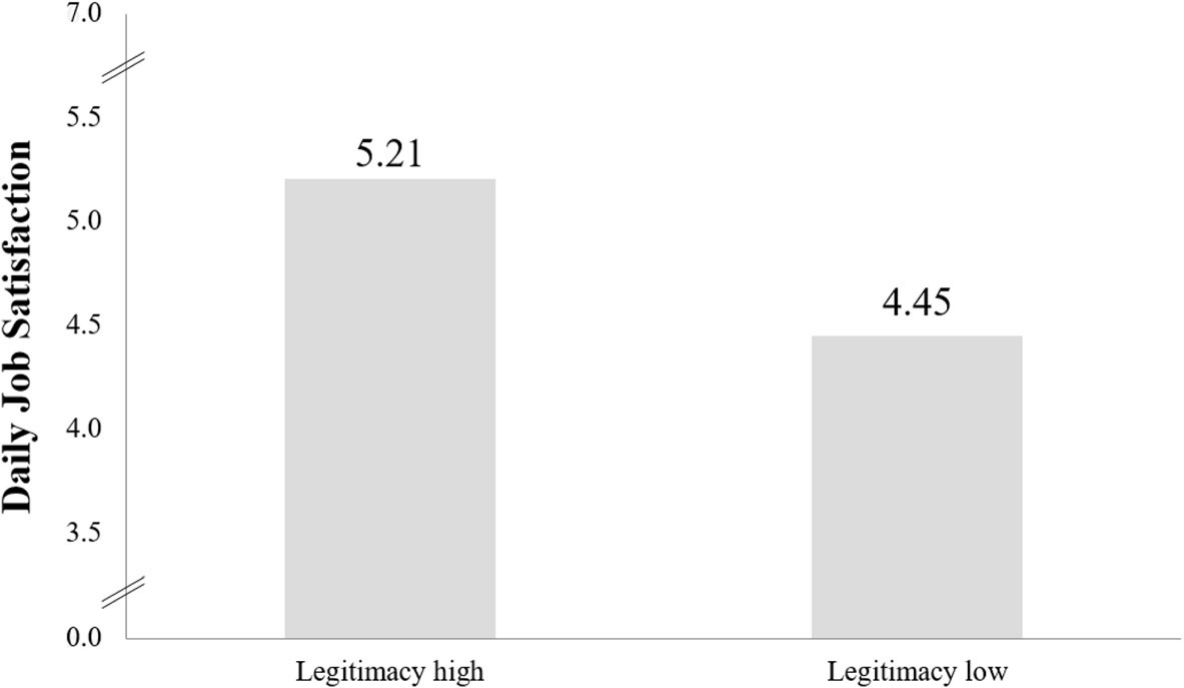
Perceived legitimacy of administrative work and daily job satisfaction

The overall mean for daily job satisfaction was 4.87; differences between positions were not significant, although values for residents were comparatively low (4.49). Daily job satisfaction was not significantly related to the sheer amount of administrative work. However, the extent to which the surgeons saw administrative work as being their proper duty and not keeping them from important medical work (i.e., as legitimate) was significantly associated with job satisfaction (*r* = .467). The corresponding difference in job satisfaction between surgeons with high (*n* = 38) versus low (*n* = 35) judgments of legitimacy is shown in Fig. 2; Daily job satisfaction was significantly different between the two groups; t (71) = 3.68, *p* < .001.

Surgery was the most preferred task; however, job satisfaction was not significantly correlated with hours spent in surgery (*r* = .13); it was, however, correlated with the extent to which doing surgery was experienced as positive (*r* = .418, *p* < .001); it was also associated with the extent to which surgeons were satisfied with the responsibility they could assume during surgery (*r* = .408, *p* = .001). Thus, a positive experience with the core task contributed substantially to satisfaction.

This satisfaction with one’s role in surgery is clearly related to position: Residents were significantly less satisfied with the responsibility granted to them (3.86 on a scale from 1 to 5) than fellows/chief residents (4.42) and chiefs/consultants (4.79). A similar difference was found in the general questionnaire, where residents indicated significantly less satisfaction with training (e.g., “training in medical specialization is well supported in our ward” [21]), with a mean value of 3.18 on a five-point scale (chiefs/consultants: 4.0; fellows/chief residents: 3.81).

## Discussion

### Main results and comparability to existing studies

Regarding daily tasks, we found that a little more than one fifth of the time (21.2%) was spent with tasks that are directly surgery related (i.e., surgery and preparation for surgery). Another fifth (21.7%) was directly patient-related; about 10 % (10.4%) was spent in meetings and communicating about patients, and somewhat less than one fifth (18.6%) with documentation and administration. The remaining time was spent with teaching, research, leadership and management and not task-related activities.

There are few other studies that analyzed the workday routine of physicians working in hospitals, and only the study by Mache et al. [23] is reasonably similar to ours. Some other studies either had much fewer surgeons [24], or a much smaller amount of work time analyzed [25]. Mache et al. [23] observed 20 residents for 60 work days, collecting 576 h of data. To compare their results with the current study, we combined surgery and preparation for surgery into surgery-related tasks; consultation hours, ward rounds, patient related discussions and calls into individual patient work; reports and patient related conferences into meetings and team communication about patients; writing patient related records and administration into documentation and administration. As participants in Mache et al. all were residents, we compare their data with those from our residents. The residents in the study by Mache et al. spent somewhat more of their time (24.4%) with surgery-specific work, as compared to 17.34% for our residents. The percentage spent on individual patient work was comparable (Mache et al.: 21.69%; residents in our study: 23.64%), and so was the percentage of time spent for documentation and administration (Mache et al.: 25.28%; residents in our study: 25.67%), and for teaching (Mache et al.: 1.97%; residents in our study: 1.93%). The only really large difference was observed for meetings and team communication about patients, which was 23.6% in the study by Mache et al. and 10.86% for the residents in our study. As the categories could not be matched exactly, these data suggest a reasonable convergence overall.

In terms of attractiveness, surgery was by far the most attractive task, confirming surgeons’ passion for their core tasks [1, 2]. Their core role is attractive to them, and this is true for all hierarchy levels; it therefore is not surprising that doing surgery is a source of satisfaction. However, it is not simply the amount of time spent with surgery that counts; it is experiencing surgery as positive and having the aspired responsibility during surgery that is associated with daily satisfaction. By contrast, writing patient reports and doing administrative work constitute the least attractive tasks, and the extent to which these tasks are considered illegitimate in the sense that they are not part of one’s role and detract from medical tasks proper is associated with lower daily satisfaction. Interestingly, attractiveness of doing research did not correlate with attractiveness of the other tasks. Although this result is based on the small subsample of those who are in an environment involved in research and therefore can only be interpreted tentatively, it suggests that doing research is not perceived as an integral part of the surgeon role. Thus, whereas it is hard to imagine someone to become a surgeon who is not interested in patients and in surgery, choosing surgery as a profession may not tell us much about this person’s interest in doing research.

Typically, when differences between positions occurred, they indicated lower satisfaction among the residents. Residents spent more time writing patient reports and doing administrative work than the other groups, and they rated these tasks as less attractive than surgeons in other positions. Furthermore, they were less satisfied with the responsibility granted to them during surgery. This reduced responsibility may well be justified by their less advanced level of training; however, it may also reflect problems with training and coaching, which were rated as less satisfying by residents in the general questionnaire. However, it is also possible that the lower job satisfaction corresponds to the U-shaped association of job satisfaction with age. Job satisfaction has been found to decline in early career stages, followed by an increase later on; more experienced physicians have been found to be more satisfied in several studies, possibly due to greater autonomy and responsibility, but also to lower private demands (e.g., small children) and greater skills in coping with high demands [26].

Regarding gender, there were not many significant differences, but those that did occur usually implied better values in terms of liking tasks and satisfaction for female surgeons. We have no immediate explanation for this finding.

### Strengths and limitations

There are several limitations of this study. First, all data are based on self-report, which entails the danger of common method bias. Using daily reports attenuates this bias, as they reduce the tendency to accommodate recalled events to pre-existing beliefs and attitudes. Furthermore, job satisfaction was not correlated with the amount of time spent in surgery but only with appreciation of surgery; this result indicates that participants did not let factual reports be colored by their attitudes but clearly distinguished between facts and their evaluation. Thus, although we cannot rule out a common method effect, it does not seem to strongly distort our results. A second limitation is the rather modest sample size. The fact that our results corresponded reasonably well with those of other studies is encouraging in this respect, but further research is needed on these issues.

Second, the sample size is not very high, especially when breaking down results by subgroups. On the other hand, diary data about hospital surgeons’ workday are rare; responding to daily questionnaires is cumbersome, and motivating surgeons to participate is not easy. Typically, therefore, sample sizes in other studies that used a similar approach are lower than those in our study [23, 24]. Results based on such sample sizes can only be considered suggestive; on the other hand, they provide detailed information that most other studies cannot provide.

Using single-item measures represents another limitation, as scales with more items would have been preferable. However, more items also increase the danger of people dropping out. Research has increasingly shown that single-item measures often are acceptable [27, 28]; for instance, the validity of single items has repeatedly been shown for job satisfaction [29]. Considering the additional burden of many items and the corresponding danger of people not participating or dropping out, it therefore seems justifiable to use single-item measures.

Using reports of daily activities over 5 days constitutes a strength of our study; it reduces method bias (see above), and it provides insights into daily tasks that are being carried out and into the attitudes of the surgeons concerning these activities.

### Practical importance and future implications

Possible consequences might relate to the way work is organized. Some of the tasks that physicians resented refer to work they should not have to do, such as spending a lot of time getting access to information that should be readily available; to organize beds for patients, etc. A focus on reorganizing work in a way that reduces non-medical demands on physicians would reduce their workload, most notably potentially illegitimate tasks, and at the same time increase the percentage of work that is related to their core role, which also are the tasks they like best. Some tasks may simply be redundant (see [30]), others, such as dealing with insurance companies, but also documentation tasks, might be taken over by specially trained nurses or administrative staff (see [30]). Programs employing scribes, who take over much of documentary tasks, have been shown to have positive effects [31], including reduced physician pre-session and post-session time and time spent in visits, while increasing patient satisfaction [32] and efficiency [33]. Furthermore, improving the usability of electronic health records (EHR) deserves attention [31].

We mentioned above that residents were occupied with more tasks that were judged not very attractive, and that they tended to be less satisfied, not least with regard to the responsibility granted to them. It is difficult to judge to what degree this comparatively low satisfaction is fueled by unrealistic aspirations, for instance in terms of underestimating the time it takes to acquire expertise, and thus attributing slow progress to inadequate coaching and training opportunities. Nevertheless, it might well be related to training issues, which also were reported to be prominent in the study by Seelandt et al. [2]. It is possible that training in some hospitals might be planned and executed in a more systematic manner and that newer training methods might be utilized to a greater degree to ensure optimal training and coaching (see [34]).

## Conclusions

Golder et al. [35] concluded for hospital doctors in general that they are highly motivated despite growing time and effort for administrative work; this conclusion can also be drawn for the hospital surgeons participating in our study.

The proportion of core tasks (i.e., surgery-related tasks) to other tasks, most notably administrative tasks, remains a concern. As Becker et al. [36] note, administrative tasks are associated with “the feeling that administrative requirements are nonmedical tasks and keep the doctors from doing their originally assigned work (p. 100)”; see also [16]. Obviously, administrative tasks are found in any job. But as their proportion grows, they are increasingly perceived as illegitimate, and associated with lower satisfaction. Our results are in line with studies showing that illegitimate tasks are associated with various types of stress symptoms [22, 37, 38].

Given the difficulties to attract medical students and residents to surgery [39], but at the same time the dedication for surgery work proper displayed by practicing surgeons [4], thinking about measures to increase the proportion of time spent doing surgery, to decrease the amount of administrative work, to optimize training and development for young surgeons, and thus to create conditions in which surgeons can find fulfillment through being involved in high quality surgery, is likely to benefit them as well as their patients.

## Additional file

Additional file 1: Questionnaire items specifically developed for this study, in English (DOCX 30 kb)

## Data Availability

The datasets used and/or analyzed for the current study are available from the corresponding author on reasonable request.
