# Supplementary material for: The workday of hospital surgeons: what they do, what makes them satisfied, and the role of core tasks and administrative tasks; a diary study
Source: BMC Surg. 2019 Aug 14;19:112. doi: 10.1186/s12893-019-0570-0 (PMC6694625; doi:10.1186/s12893-019-0570-0)
Supplement: Supplementary file 1 — Questionnaire items specifically developed for this study, in English (DOCX 30 kb) [file 12893_2019_570_MOESM1_ESM.docx]

Holzer, E., Tschan, F., Kottwitz, M. U., Beldi, G., Businger, A. P., & Semmer, N. K. (2019).

The workday of hospital surgeons: What they do, what makes them satisfied, and the role of core tasks and administrative tasks; a diary study.

*BMC Surgery*, doi 10.1186/s12893-019-0570-0

**The workday of hospital surgeons:**

**Self- developed questionnaires**

**Job Satisfaction**

General Survey

How satisfied are you if you consider your work situation as a whole?

|  |  |  |  |  |  |  |  |
| --- | --- | --- | --- | --- | --- | --- | --- |

|  | | ➀ | ➁ | ➂ | ➃ | ➄ | ➅ | ➆ |  |
| --- | --- | --- | --- | --- | --- | --- | --- | --- | --- |
|  | exceptionally dissatisfied | | very dissatisfied | quite dissatisfied | neither- nor | quite satisfied | very satisfied | exceptionally satisfied | |

Daily Questionnaire

Regarding my situation at work overall, at this moment I am …

|  |  |  |  |  |  |  |  |
| --- | --- | --- | --- | --- | --- | --- | --- |

|  | | ➀ | ➁ | ➂ | ➃ | ➄ | ➅ | ➆ |  |
| --- | --- | --- | --- | --- | --- | --- | --- | --- | --- |
|  | exceptionally dissatisfied | | very dissatisfied | quite dissatisfied | neither- nor | quite satisfied | very satisfied | exceptionally satisfied | |

**Daily Activities**

Now we would like to know which activities you carried out in the last 24 hours. For each activity, please indicate whether or not you carried it out in the last 24 hours.

| List of tasks | Did you carry out the task? | | | How much time did that take in total? | | How did you experience this activity? | | | | | Was it necessary and reasonable for YOU to carry out this task? | | | | | |
| --- | --- | --- | --- | --- | --- | --- | --- | --- | --- | --- | --- | --- | --- | --- | --- | --- |
|  | no | only once | several times | Hours | Minutes | very negative | nega­tive | neutral | posi­tive | very posi­tive | absolutely not | no | neither - nor | yes | very much |  |
| **Daily meetings** | □ | □ | □ | __ | __ | ➀ | ➁ | ➂ | ➃ | ➄ | ➀ | ➁ | ➂ | ➃ | ➄ |  |
| **Ward rounds** | □ | □ | □ | __ | __ | ➀ | ➁ | ➂ | ➃ | ➄ | ➀ | ➁ | ➂ | ➃ | ➄ |  |
| **Consultations, entry examination, postoperative care** | □ | □ | □ | __ | __ | ➀ | ➁ | ➂ | ➃ | ➄ | ➀ | ➁ | ➂ | ➃ | ➄ |  |
| **Preparing surgical strategy** (Reading; discussing and planning the procedure etc.) | □ | □ | □ | __ | __ | ➀ | ➁ | ➂ | ➃ | ➄ | ➀ | ➁ | ➂ | ➃ | ➄ |  |
| **Performing surgery / assisting surgery** | □ | □ | □ | __ | __ | ➀ | ➁ | ➂ | ➃ | ➄ | ➀ | ➁ | ➂ | ➃ | ➄ |  |
| **Patient documentation** (operation reports, case history, entry and discharge reports, medical certificate) | □ | □ | □ | __ | __ | ➀ | ➁ | ➂ | ➃ | ➄ | ➀ | ➁ | ➂ | ➃ | ➄ |  |
| **Patient related conversations / telephone calls** (with general practitioners, relatives etc.) | □ | □ | □ | __ | __ | ➀ | ➁ | ➂ | ➃ | ➄ | ➀ | ➁ | ➂ | ➃ | ➄ |  |
| **General Administration** (service billing, quality assurance, forms,  signing up patients for specific examinations, other administration) | □ | □ | □ | __ | __ | ➀ | ➁ | ➂ | ➃ | ➄ | ➀ | ➁ | ➂ | ➃ | ➄ |  |
| **Patient-related meetings** (case discussions, meeting for consultation, mortality and morbidity conferences, etc.) | □ | □ | □ | __ | __ | ➀ | ➁ | ➂ | ➃ | ➄ | ➀ | ➁ | ➂ | ➃ | ➄ |  |
| **Specific meetings** (e.g., multidisciplinary tumor conferences, X-ray conferences, etc.) | □ | □ | □ | __ | __ | ➀ | ➁ | ➂ | ➃ | ➄ | ➀ | ➁ | ➂ | ➃ | ➄ |  |
| **Continuing education and training** (conferences, reading, attending lectures, etc.) | □ | □ | □ | __ | __ | ➀ | ➁ | ➂ | ➃ | ➄ | ➀ | ➁ | ➂ | ➃ | ➄ |  |
| **Research, publishing** (including preparation of presentations) | □ | □ | □ | __ | __ | ➀ | ➁ | ➂ | ➃ | ➄ | ➀ | ➁ | ➂ | ➃ | ➄ |  |
| **Teaching** (preparing lessons; instruction, introduction of new employees) | □ | □ | □ | __ | __ | ➀ | ➁ | ➂ | ➃ | ➄ | ➀ | ➁ | ➂ | ➃ | ➄ |  |
| **Leadership and Management** | □ | □ | □ | __ | __ | ➀ | ➁ | ➂ | ➃ | ➄ | ➀ | ➁ | ➂ | ➃ | ➄ |  |
| **other** (please specify  _________________________ | □ | □ | □ | __ | __ | ➀ | ➁ | ➂ | ➃ | ➄ | ➀ | ➁ | ➂ | ➃ | ➄ |  |

**Legitimacy of administrative work** (daily questionnaire)

Do you think the demand for administrative work in the last 24 hours overall…

|  | Not true |  |  |  | Very true |
| --- | --- | --- | --- | --- | --- |
| … was adequate? | ➀ | ➁ | ➂ | ➃ | ➄ |
| … kept you from important medical activities? | ➀ | ➁ | ➂ | ➃ | ➄ |
